# Age-Friendly Care, PA: A Geriatric Workforce Enhancement Program

**DOI:** 10.1093/geroni/igab046.1225

**Published:** 2021-12-17

**Authors:** Erica Husser, Donna Fick, Judith Hupcey, Marie Boltz, Lisa Kitko

**Affiliations:** 1 Penn State Ross and Carol Nese College of Nursing, Spring Mills, Pennsylvania, United States; 2 Pennsylvania State University, University Park, Pennsylvania, United States; 3 Pennsylvania State University, Pennsylvania State University, Pennsylvania, United States

## Abstract

Age-Friendly Care, PA is co-led by Primary Health Network, the largest Federally Qualified Health Center in Pennsylvania, and Penn State College of Nursing that aims to bring reliable, high-quality, age-friendly care to all older adults living in rural PA. Sponsored by HRSA through its Geriatric Workforce Enhancement Program, Age-Friendly Care, PA utilizes the ECHO, all-teach-all-learn, platform to engage isolated rural providers in incorporating the 4Ms (IHI) into their practice. Age-Friendly Care, PA reaches out directly to rural older adults and their care partners to co-design education and support. We have hosted 28+ events and reached 450+ individuals. Results include tracking and improvement in quality indicators assessed including support for individuals living with dementia and their care partners (NA-66.7%), risk for opioid misuse (NA-78%), high-risk medication management (NA-47.8%), fall-risk management (NA-9.4%), and advanced care planning (NA-8.9%). We will discuss the creation, co-development, implementation, lessons learned, and future of Age-Friendly Care, PA.

